# Conversion of savanna rangelands to bush dominated landscape in Borana, Southern Ethiopia

**DOI:** 10.1186/s13717-016-0049-1

**Published:** 2016-05-04

**Authors:** Teshome Abate, Ayana Angassa

**Affiliations:** School of Animal and Range Sciences, Hawassa University, Hawasa, Ethiopia

**Keywords:** Bush encroachment, Landscape pattern, Remote sensing, LULC, Borana rangeland

## Abstract

**Introduction:**

Analyzing trends of land use systems and the changes occurred overtime is an effective way of assessing the impacts of land use/land cover (LULC) changes on ecosystem function. It provides important insights for understanding the spatial patterns of land use processes. The rangelands of southern Ethiopia are adversely affected by increased human population pressure, encroachment of crop cultivation, and bush encroachment. Hence, it is vital to understand the trends of rangeland vegetation cover dynamics.

**Methods:**

This paper evaluates land use/land cover changes and spatial patterns between 1987 and 2003 in Yabelo (5426 km^2^), Borena rangelands of southern Ethiopia. We used a combination of three Landsat Thematic Mapper (TM) 1987, Landsat TM 1995 and Enhanced Thematic Mapper Plus (ETM+) 2003, and local perceptions. A pixel-based supervised classification with maximum likelihood classifier was used to classify images. The accuracy of classification was assessed for 1987 (81.8 %), 1995 (84.6 %), and 2003 (81.3 %).

**Results:**

The results showed that the Borana rangelands had undergone substantial changes during the last 16 years. Between 1987 and 2003, we observed a considerable increase in woodland cover (11.7 %), bushland cover (17 %), cultivated land (72.5 %), and settlements (79.8 %). The results showed a rapid decline in grassland cover (7.7 %), shrubby grassland cover (86 %), and bareland (0.7 %). The spatial pattern analysis indicate that the Borana rangeland was fragmented and characterized by the proliferation of large numbers of patches with a decline in patch index, increased patch density, and irregular shape of patches within a landscape. Local communities’ perceptions indicate that recurrent drought, increased human population size, and expansion of cultivation were largely responsible for the observed LULC changes in the study area.

**Conclusions:**

LULC changes contribute to rangeland degradation and weaken the traditional practices of rangeland management. We suggest appropriate management measures to halt the impact of disturbances on LULC dynamics and its implication on the livelihoods of the Borana pastoralists.

## Introduction

Understanding the complexity of impacts of land use/land cover (LULC) changes, landscape patterns, their driving forces, and impacts on human and ecological processes is crucial for designing appropriate natural resource management and decision-making processes. Analyzing landscape patterns and the changes overtime is an effective way of assessing the impacts of LULC change on ecosystem function. It also provides an important insight in understanding the spatial patterns in relation to land use processes (Lafortezza et al. 2010). According to Lu et al. (2004), LULC changes are a dynamic and complex process depending on the existing socio-economic and biophysical conditions. For example, landscape patterns indicate the complex interaction between the ecological and anthropogenic variables (Wu et al. 2011). Usually, anthropogenic activities have the potential to alter a landscape structure and ecological function of the landscape overtime (Long et al. 2010; Morris 2010). Thus, understanding the changes in a given landscape may allow researchers to explore the impact of human activities on ecological systems (Teixido et al. 2010). This is achieved by examining a variety of landscape matrices that reveal landscape structure (Long et al. 2010). Landscape matrices is useful in assessing the spatial structure of a land cover in terms of number, size, shape, and configuration of patches of the different LULC classes (McGarigal et al. 2002).

Kamusoko and Aniya (2006) have shown that analysis of LULC and landscape structure is useful in understanding the extent and implications of fragmentation within landscapes. Some of the effects of fragmentation on landscape structure are reduction in the size of habitats, largest patch index, and patch size and increase in the number of patches and isolation of habitat patches (Herold et al. 2003; Turan et al. 2010). The largest patch index indicates the largest patch in each land cover class in a given area (McGarigal et al. 2002). A decrease in the largest patch index signifies an increase in fragmentation and hence an increase in degradation. The number of patches is a measure of landscape configuration. It represents the spatial character of a class or a landscape and indicates the extent of fragmentation. In a given landscape, mean patch size affects biomass, productivity, nutrient storage, composition, and diversity of species (Forman and Godron 1986) and the populations of animals that landscape can support, which can also serve as an indicator of landscape function and fragmentation (Bender et al. 1998). The mean shape index is a measure of patch complexity (McGarigal et al. 2002). Simple patches shapes such as circles and squares have different effects on ecological functions and human management than more complex ones. A patch shape can affect the size and nature of patch interaction within a given environment, mainly through the edge effect phenomenon, which indicates changes in microclimate, disturbance factors, and the ecological processes at patch level (Leitao and Ahern 2002). As shape complexity increases, so does edge habitat abundance (Sidiropoulou et al. 2015). Overall, FRAGSTAT, which is a spatial pattern analysis program to quantify landscape structure, has been widely used by several scholars (McGarigal and Marks 1994; Li et al. 2001; DiBari 2007; Sidiropoulou et al. 2015).

The Borana rangelands in southern Ethiopia comprise important cultural landscapes with a unique feature of the *tula*-well landscapes^1^ (Angassa and Oba 2007). Until few decades ago, the Borana rangelands are considered as one of the most productive and resilient ecosystems in East Africa (Hogg 1997). In recent years, however, the Borana rangelands have greatly reduced in terms of grassland cover as a result of anthropogenic and naturally induced factors (Coppock 1994; Solomon et al. 2006b; Angassa and Oba 2008b) with consequences on the livelihood of the local communities. The conversion of the savanna rangelands of Borana to bushland and cropland had began around the early 1970s due to the official ban of fire (Coppock 1994) followed by the expansion of bush encroachment and crop cultivation in the 1980s (Angassa and Oba 2008b). During that time, about 40 % of the Borana rangelands were affected by bush encroachment (Coppock 1994), which result in large declines among the valuable perennial grasses and grass species diversity (Angassa et al. 2012a).

Changes in LULC in the Borana rangelands have had substantial impacts (e.g., shrinkage of grassland, bush encroachment, and rangeland degradation) on the rangeland resources and ecosystem services (Oba et al. 2000). Earlier studies (e.g., Borghesio and Giannetti 2005; Homann et al. 2005) have argued that LULC change has the potential to cause a wide range of environmental problems including bush encroachment, overgrazing, and soil degradation. The ban of fire and drought is the most important factor in driving LULC dynamics in the Borana rangelands of southern Ethiopia (Oba 1998). Recurrent drought caused decline pasture and water, increased depletion of herds through mortalities, forced off take (Desta and Coppock 2002), reduce household income, local food insecurity, and poverty (Tache and Oba 2010), and this led change in pastoral land use. Recent studies (Angassa and Oba 2008b) have indicated ban on the use of fire increased bush encroachment. Hence, exploring land use patterns in arid and semi-arid rangelands of southern Ethiopia is essential to understand its impact on ecological resources (e.g., a reduction in grass cover and species diversity of palatable herbaceous plant species) and processes such as bush encroachment and rangeland fragmentation in the region. Widespread bush encroachment and increased grazing pressure around water points and settlements, as well as intensified cultivation had resulted in the loss of biodiversity (Oba 1998; Oba et al. 2000), altered soil nutrient contents such as nitrogen, phosphorous, organic carbon, and cation exchange capacity (Angassa et al. 2012b) and potentially loss of resilience in the system. The Borana pastoralists depend mostly on livestock production where livestock feed resources are largely constrained by frequent drought and changes in LULC patterns (Desta and Coppock 2002; Angassa and Oba 2007). The old ecological balance of the Borana rangeland has severely affected by the expansion of bush encroachment, cultivation, land tenure changes (e.g., the establishment of semi-private resource tenure in the rangelands such as ranch and enclosure), and sedentarization of pastoral community (Angassa and Oba 2008b). As a result, the Borana rangeland may keep on experiencing dramatic changes in LULC over the next several decades (Haile et al. 2010; Elias et al. 2015).

Given the impact of LULC changes on savanna ecosystem in Borana and the uncertainty of future driving forces of LULC changes, analysis of the potential changes in terms of land use pattern is needed to overcome any negative impacts on the environment. Hence, analysis of the magnitude of LULC changes and its driving forces may provide key environmental indicators for scientific research, resource management, and designing future development strategies (Lu and Weng 2007). Analysis of LULC changes has also been used as an input for a range of environmental models and policy-driven needs (Rosenqvist et al*.*^2003^). Furthermore, information on LULC changes may play a decisive role in the management of savanna ecosystems (Duadze 2004) and biodiversity monitoring (Aynekulu et al. 2008). Even though previous studies (Oba 1998; Coppock 1994; Homann et al. 2005; Solomon et al. 2006b; Angassa and Oba 2008a; Tache and Oba 2010) have reported some aspects of LULC changes in Borana, those studies often deal with quantifying LULC changes and there causes using socio-economic survey or exclusively based on field observation. Yet, information related to LULC changes and landscape pattern analysis using satellite images has been rarely reported in the Borana rangelands of southern Ethiopia. Therefore, concrete information on LULC changes in connection with their underlying causes at spatial and temporal scales is needed. Therefore, the aim of this study was to facilitate understanding of the patterns of LULC changes over between 1987 and 2003, the main drivers of changes in the arid and semi-arid rangelands of Borana in southern Ethiopia. The following questions were used to address the objective of the study: (1) what are the main LULC types and extent of change in Borana rangelands? (2) what are the underlying causes of LULC changes and associated consequences?

## Methods

### Study area

**Fig. 1:**
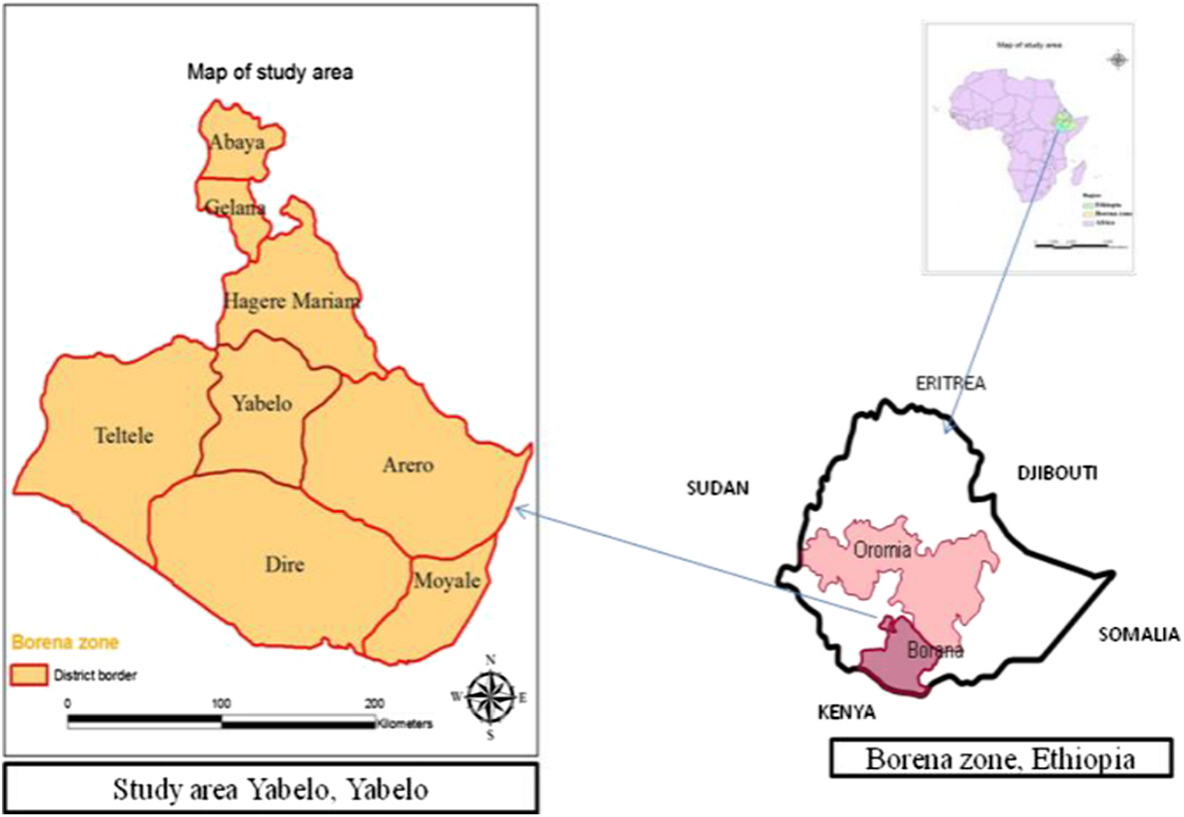
Map of the study area in Yabelo district southern Ethiopia

The study was conducted in the Borana rangelands of southern Ethiopia, Yabello district. The district covers a total area of 5426 km^2^. The northern part of the Borana rangeland is bordered by the neighboring highlands of Borana zone of Oromia Regional State of Ethiopia. The southeastern part is bordered by Somalia, while the southern limit is bordered by Kenya (Fig. 1). It is located between latitude 4° 30′ 55.81″ and 5° 24′ 36.39″ N and longitude 7° 44′ 14.70″ and 38° 36′ 05.35″ E. Generally, the altitude of the Borana rangelands is within the range of 1000–1500 m above sea level (m.a.s.l) with few hills up to 2000 m.a.s.l (Coppock 1994). The climate of the Borana rangeland is mostly arid and semi-arid. The inter-annual rainfall was varied with an average annual rainfall of 526.75 mm. The rainfall pattern is bimodal, with 60 % of the annual rainfall occurring between March and May (main rainy season) followed by a short rainfall between September and November (Dalle et al. 2006). The average annual temperature of the area varies from 19 to 26 °C. Drought is common in the study area every 5 to 6 years (Desta and Coppock 2002). The Borana pastoralists had a well-established water and grazing lands management system (Dalle et al. 2006). The vegetation of the study area is a tropical savanna with varying proportions of open grassland and perennial herbaceous and woody vegetation. The Borana rangeland composed of *Acacia-Commiphora* small-leaved deciduous woodlands, with a mixture of the genera *Acacia*, *Boswellia*, and *Commiphora* (Coppock 1994). The region is characterized by the limitation of surface water where the major source of water for humans and livestock are from wells and ponds. Soils of the study area were derived from ancient alluvial and volcanic materials (FAO 1986). Generally, soils are classified as shallow red sandy loam in the uplands and vertisols in the bottomlands (Dalle et al. 2006). The livelihood of the Borana pastoralists has been heavily dependent on cattle production, and the region is also one of the most important areas of cattle production in Ethiopia. Grasslands have the main source of feed for cattle.

### Data type and image analysis

Two Landsat Thematic Mapper 5 (TM) of 30 m resolution for the 1987 and 1995 (images acquired on February 9, 1987 and January 30, 1995 with sensor characteristics of path/raw 168/056 and 168/057) and Landsat Enhanced Thematic Mapper plus (ETM+) for 2003 (images acquired on January 12, 2003 with path/raw 168/056 and 168/057) with a spatial resolution of 30 m were acquired from the of the Global Land Cover Facility (GLCF) archive (USGS 2009). The imageries used in this study were cloud-free, dry season, and the selection of imageries was depending on their availability. Moreover, topographic map (at a scale of 1:50000), which covers the entire study area was obtained from the Ethiopian Mapping Agency, road network, district boundary, protected area map, Google earth map, meteorological data, two land use maps (maps acquired from the International Livestock Research Institute (ILRI) GIS services web page and the Ethiopia Biomass Inventory Planning Project) were used as supplementary ancillary data for classification and accuracy assessment. Furthermore, about 450 GPS ground truth data that consists of 230 for 2003 map, 124 for 1995, and 96 for 1987 map were gathered and used for validation. The data on ground truth were collected in consultation with the local people regarding the history of land use and driving forces of changes. The Landsat TM and ETM+ images used in this study were orthorectified by the image supplier to Universal Transverse Mercator (UTM) projection on the WGS 84 datum, UTM zone 3. The geometric correction and image-to-image registration was done by taking the 1987 image as a reference image. The 1995 image was co-registered with the 2003 image with a root mean square of less than 0.5 pixel. Since a single image had not been covering the spatial extent of the entire study area, two scenes of images were used to mosaic on a band-by-band basis and sub-setting was made. The pre-processing (e.g., haze reduction, linear stretching, and histogram matching) following the procedures recommended in ERDAS (1999) was used to enhance visualization and interpretation. The images were resampled to 30 m. Finally, the false color composite use of band combination of RGB 4, 3, and 2 were used for the 1987 and 2003 images, whereas RGB 7, 4, and 2 band combinations were used for the 1995 image. The nomenclature of LULC classes used in this study were adopted from the classification scheme used by previous studies (Haile et al. 2010; Tsegaye et al. 2010) in arid rangelands of North-eastern Ethiopia. Land class nomenclatures were modified into seven classes, namely: woodland (land with woody species cover >20 %, height of plant ranges from 5 to 20 m, areas with trees mixed with bushes and shrubs, with little use especially for cattle. It includes those sites where woody cover is fully mature and herbaceous plants have been almost eliminated); bushland (interpreted in terms of land with >20 % bush or shrub cover <5 m in height); shrubby grassland (this unit consists the former grassland sites where shrubs and bush have increased in density to be co-dominated with herbaceous plants in terms of cover); grassland (consists of grassland with <20 % bush or shrub cover, grass and herb cover with scattered trees and shrubs, areas with permanent grass cover used for livestock grazing including the communal and protected areas. They also tend to be open areas with good visibility on flat areas and hill slopes, while another descriptive term would be mixed savanna); cultivated land (this unit includes cropping area); bareland (refers to an area with no vegetation, which occur in rangelands including gullies and exposed rocks); and settlement (comprises urban and rural settlements in the study area) (Haile et al. 2010; Tsegaye et al. 2010).

### Image classification and accuracy assessment

Classifications of imageries were done using both unsupervised and supervised classification methods. The results of unsupervised classification procedure was used as a guide for the selection of training sites for supervised classification, and it also provided a preliminary information about the potential spectral clusters to be assigned to thematic classes. Then, images were classified at pixel level using supervised image classification with maximum likelihood classifier algorithm. As postclassification enhancement such as filtering of the final classification results reduce the heterogeneity of the classified image, filtering was not carried out in this study. This is because filtering might mislead by generalizing the classification to the extent that some mapping units for instance cultivated area, settlements, and bareland covers could be underestimated. Duadze (2004) have observed a similar experience in the arid and semi-arid savanna ecosystem of Ghana.

### Accuracy assessment

Accuracy assessments of maps were determined using error matrix and Kappa statistic (Congalton and Green 1999). Unfortunately, there was no high-resolution satellite imagery or aerial photographs available to assess the accuracy. The validation was done using ground truth data (GPS points 80, 132, and 230), which were gathered during the field work in reference to 1987, 1995, and 2003 maps, respectively. At each ground truth point, discussions were held with the local elders who were familiar with LULC classes to recall about LULC history covering the 1987, 1995, and 2003 periods. Furthermore, to understand the dynamics of LULC, the possible drivers and consequences of the changes were explored using group discussions with local elders and key informants (i.e., agricultural development workers and researchers). The discussion questions were open ended to identify historical events and linkages to the land cover change. Furthermore, published documents and public statistics were used to document the major cause and associated consequences.

### Land cover change analysis

LULC change was done using postclassification comparison method where Landsat image for each year was classified and labeled independently, and then comparison was made using an overlay procedure (Lu et al. 2004). In connection with this, the net change, persistence, and the net change-to-persistence ratio (Pontius et al. 2004; Braimoh 2006; Tsegaye et al. 2010) were computed to show the resistance and vulnerability of a given LULC type in IDRIS ANDES software of Land Change Modeler. The magnitudes of change in terms of LULC were determined using the following variables: total area (TA), changed area (CA), change extent (CE), and annual rate of change (CR). The variables were calculated as follows (Addis 2010):

$$ \mathrm{C}\mathrm{A} = \mathrm{T}\mathrm{A}\ \left({t}_2\right)\hbox{-} \mathrm{T}\mathrm{A}\ \left({t}_1\right) $$

$$ \mathrm{C}\mathrm{E} = \left[\mathrm{C}\mathrm{A}/\mathrm{T}\mathrm{A}\ \left({t}_1\right)\right]\times 100 $$

$$ \mathrm{C}\mathrm{R} = \mathrm{C}\mathrm{E}/\left({t}_2\hbox{-} {t}_1\right) $$

where *t*_1_ and *t*_2_ are the beginning and ending time of the land cover studies conducted.

### Landscape pattern analysis

The landscape spatial pattern of the study area was determined using selected spatial matrices. Spatial matrices are algorithms used to quantify spatial characteristics of patches, (discrete areas of relatively homogeneous environmental conditions), class (aggregation of same type of patches like grassland, woodland, bareland), or entire landscape mosaics (McGarigal et al. 2002). Patches are the basic “building blocks” of landscape structure, and most matrices derive from their spatial character and distribution (Ricca and Guagliardi 2015). They differ in size, shape, and type (Forman and Godron 1986). At the class and landscape levels, some of the matrices quantify landscape composition, while others quantify landscape configuration (McGarigal et al. 2002). In our study, we adopted ten matrices for class level and ten at landscape level analysis that was also used by previous studies (Li et al. 2001; Herold et al*.*^2003^). The selected matrices describe the composition and configuration of landscape patterns. The matrices are also used to measure the landscape fragmentation, dominancy, and diversity. Then, the matrices were computed for each land cover map of 1987, 1995, and 2003 at the class and landscape levels. These matrices were determined in FRAGSTATS software 3.3 (McGarigal et al. 2002). In the analysis, we used 30-m grid resolution and the search and threshold distance of 200 m for determination of matrices at patch and class levels, respectively.

## Results

### LULC change and accuracy assessment

Table 1 provides the accuracy assessment (user’s and producer’s accuracies). The results showed that the overall classification accuracy for the 1987, 1995, and 2003 maps were 81.8, 84.6, and 81.3 %, respectively. The present results displayed a high rate of agreement between the user’s accuracy (UA) and producer’s accuracy (PA) in terms of woodland, bushland, and grassland cover changes in 1987, 1995, and 2003 (Table 1). However, the trends in the rate of agreement (*K*) between UA and PA for the changes in woodland showed a slight decline in 1995 and increased in 2003. Although *K* was constant in terms of bushland, the change between 1987 and 1995 was slightly declined in 2003 (Table 1). In terms of shifts in grassland cover, the rate of agreement showed a linear increase from 1987 to 2003 (Table 1). The changes towards shrubby grassland and bareland covers were increasing with a high rate of agreement between UA and PA towards 1995, but drastically declined in 2003 (Table 1). Overall, the rates of agreement between UA and PA in terms of cultivation and settlement areas were greatly increased in 2003 (Table 1). It seems that the spectral overlapping and similarity of some surface features were the source of misclassification in accuracy assessment. For instance, classes such as woodland and bushland covers had a closer and overlapping spectral feature in some places. Similarly, bareland, settlement, and cultivated land features after crop harvest had a closer spectral overlap in some areas, thus create a difficulty in differentiating between the different land cover classes. Furthermore, the misclassification of a class can be associated with the diversity of the various vegetation covers in the study area and use of dry season images. During the dry season, most of the natural vegetation mainly grassland and cultivated lands within a landscape may undergo dry. During the dry period, there might be high chances of land feature to be spectrally similar.

**Table 1:** Accuracy assessments for the 1987, 1995, and 2003 land use/cover maps in the Borana rangelands of southern Ethiopia

LULC cover	1987			1995			2003		
	UA (%)	PC (%)	*K*	UA (%)	PC (%)	*K*	UA (%)	PC (%)	*K*
WL	85.0	81.0	0.80	81.0	89.5	0.79	85.4	83.3	0.82
BL	90.0	81.8	0.89	83.3	69.0	0.89	80.9	82.6	0.76
GL	83.0	83.3	0.75	81.8	96.4	0.76	91.3	62.6	0.89
SGR	75.0	75.0	0.73	80.0	88.8	0.78	42.9	81.8	0.40
BAL	71.0	83.3	0.66	86.7	92.9	0.84	69.2	78.3	0.66
CUT	75.0	75.0	0.73	76.9	91.0	0.75	92.9	92.9	0.91
SET	83.3	83.3	0.82	88.0	50.0	0.81	95.0	54.3	0.94
Over all accuracy (%) 81.8				84.6			81.3		
Kappa coefficient (%) 0.77				0.82			77.8		

*WL* woodland, *BL* bushland, *GL* grassland, *SGR* shrubby grassland, *BAL* bareland, *CUT* cultivation, *ST* settlement, *UA* user accuracy, user’s accuracy represents the probability that a given pixel will appear on the ground as it is classed, *PC* (producer’s accuracy) producer’s accuracy represents the percentage of a given class that is correctly identified on the map, *K* kappa coefficient

### Magnitude of LULC changes

**Fig. 2:**
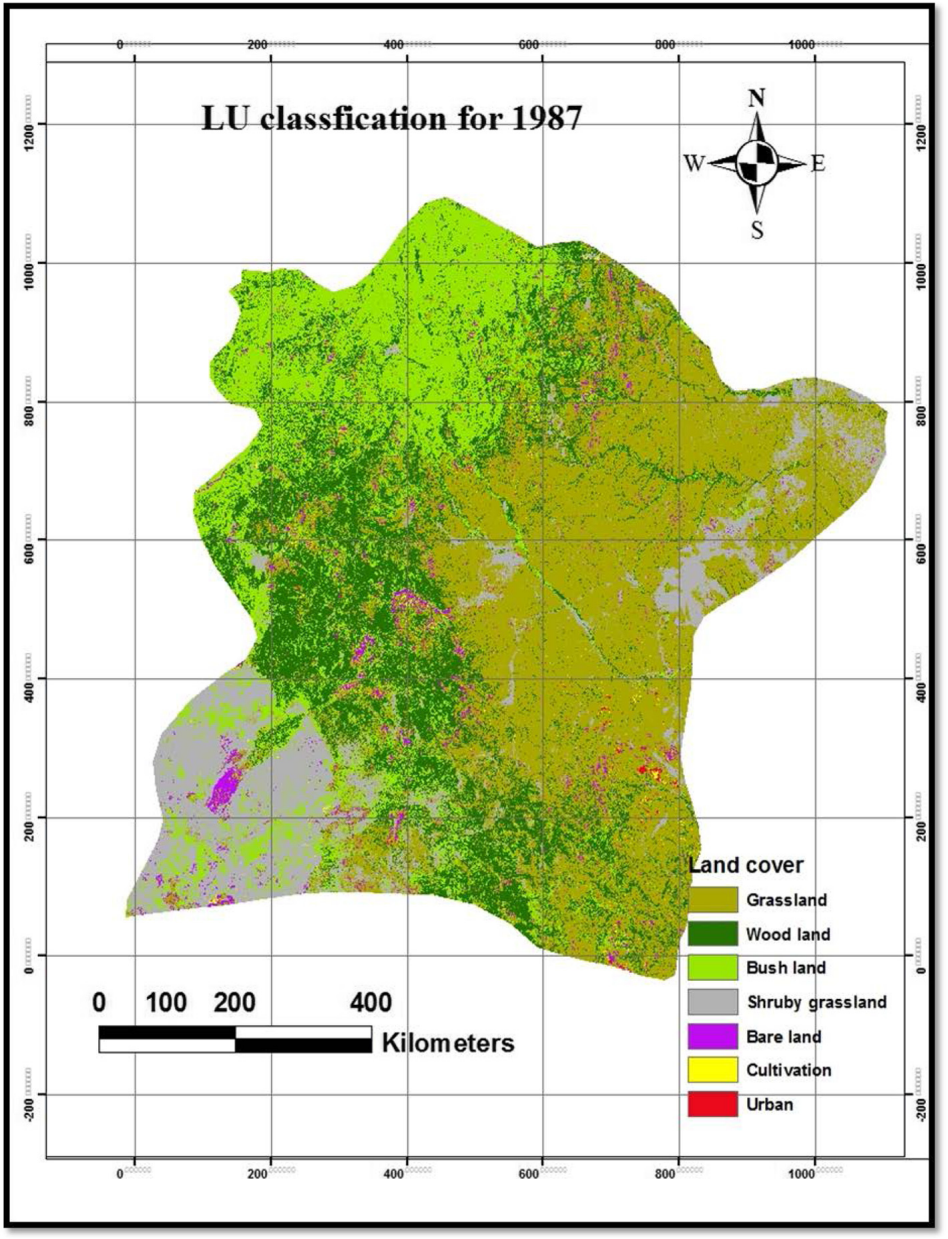
LULC change classification map of 1987 in Borana rangeland of southern Ethiopia

**Fig. 3:**
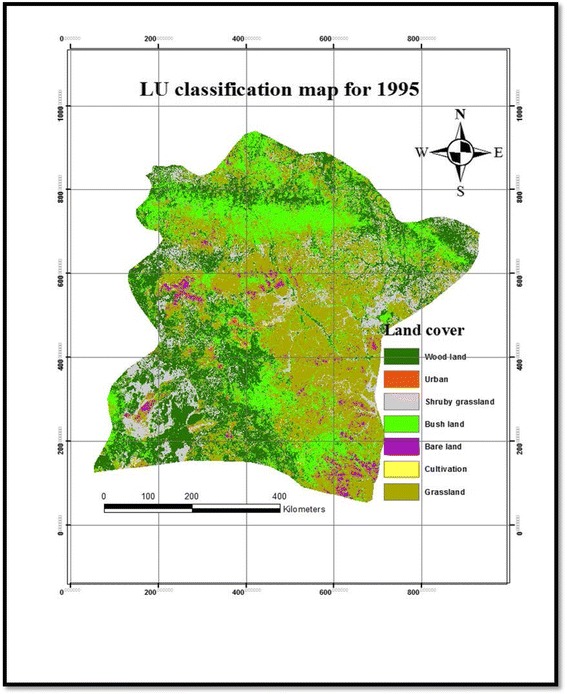
LULC change classification map of 1995 in Borana rangeland of southern Ethiopia

**Fig. 4:**
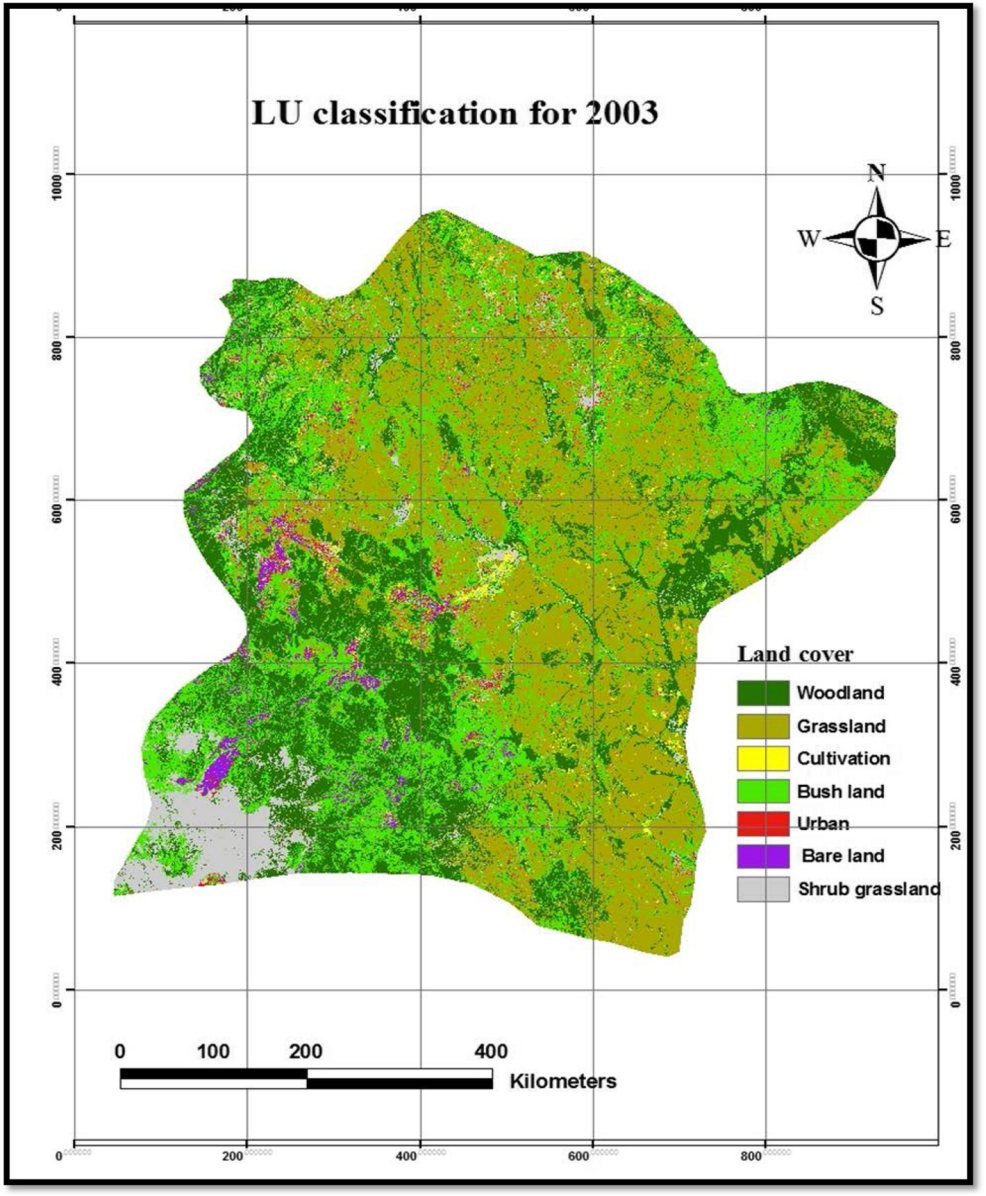
LULC change classification maps of 2003 in Borana rangeland of southern Ethiopia

Table 2 presents the magnitude of LULC changes in the Borana rangelands between 1987 and 2003. The results of our study showed that grassland cover was the dominant vegetation type in the study area between 1987 and 2003 followed by woodland and bushland (Table 2 and Figs. 2, 3, and 4). Based on the results of the present study, cultivated and settlement land covers were low (Table 2 and Figs. 2, 3, and 4). However, grassland cover showed a constant reduction between 1987 and 2003, whereas the bushland cover was rapidly expanded among the vegetation components with a change extent of 17.5 % between 1987 and 2003. Our results showed that the woodland cover was rapidly increased in the first phase of the study between 1987 and 1995 by 22.3 % and then the magnitude was greatly reduced during the second period (between 1995 and 2003) by a rate of 5.24 %, i.e., from 27.3 % cover in 1995 to 25.2 % in 2003 (Table 2). Generally, the woodland cover was increased at a rate of 11.7 % annually during the study period (Table 2). The proportion of bushland was reduced by 3 % during the first phase of the study period (1987–1995), while significantly increased by 24.8 % in the second phase (1995–2003), the grassland cover showed a rapid reduction in the earlier period (1987–1995) by 11.36 %, while it was grassland cover that was increased during the second phase of the study period (1995–2003) by 4.8 %. Generally, the magnitude of the grassland cover was reduced between 1987 and 2003 at a rate of 7.7 % annually from 40.03 % cover in 1987 to 36.75 % in 2003 (Table 2). The present result showed that the shrubby grassland cover was constantly reduced both in the earlier period (1987–1995) by 1.1 % and second phase (1995–2003) by a rate of 45.2 %. In general, the shrubby grassland was reduced at a rate of 86.1 % annually, which was the highest reduction in the landscape, from 13.1 % cover in 1987 to 7 % in 2003 (Table 2). Our results showed that there was a slight change in terms of bareland cover in the studied landscapes both in the earlier times and during the second phase with a constant magnitude of change. The extent of bareland was reduced at a rate of 0.7 % annually (Table 2). Although the size of cultivated and settlement land covers were initially insignificant, our results showed a permanent growth during the study period (Table 2). While cultivated land was substantially increased throughout the study periods, the expansion of cultivated land was higher by 111.92 % between 1995 and 2003 than the earlier period (1987–1995), which accounted for 69.52 %. Between 1987 and 2003, the extent of changes in the cultivated land was considerably increased at a rate of 72.49 % annually, from 0.3 % cover of the landscape in 1987 to 1.1 % in 2003. Likewise, settlement areas were constantly increased over the last 16 years at a rate of 79.8 % annually, from 0.2 % cover in 1987 to 1.3 % in 2003, the growth rate being higher in the second period of the study (249.34 %) than the earlier period (137 %). Over the entire study period, cultivated and settlement land covers showed a positive trend in terms of annual change, whereas the shrubby grassland displayed a negative trend. Overall, the changes in woodland, bushland, grassland, and bareland showed contrasting trends throughout the entire study period (Table 2).

**Table 2:** Area coverage and rate of LULC changes between 1987 and 2003 in the Borana rangelands of southern Ethiopia

LULC	1987 km^2^	Cover %	1995 km^2^	Cover %	2003 km^2^	Cover %	1987–1995			1995–2003			1987–2003		
							CA km^2^	CE %	CR %	CA km^2^	CE %	CR %	CA km^2^	CE %	CR %
WL	1209.5	22.3	1478.9	27.3	1369.5	25.2	269.4	22.3	2.8	−77.5	−5.2	−0.7	160,0	11.7	0.7
BL	1197.1	22.1	1161.2	21.4	1444.0	26.6	−35.9	−3.0	−0.4	288.4	24.8	3.1	252.5	17.5	1.1
GL	2172.1	40.0	1925.2	35.5	1994.4	36.8	−246.8	−11.4	−1.4	92.6	4.8	0.6	−154.2	−7.7	−0.5
SGR	713.1	13.1	705.3	13.0	379.0	7.00	−7.6	−1.1	−0.1	−318.8	−45.2	−5.7	−326.5	−86.1	−5.4
BAL	110.1	2.0	109.1	2.0	108.2	2.0	−1.1	−1.0	−0.1	0.3	0.2	0.03	−0.8	−0.7	−0.1
CUL	16.6	0.3	28.1	0.5	59.3	1.1	11.5	69.5	8.7	31.5	111.9	14.0	43.0	72.5	4.5
SET	7.9	0.2	18.7	0.4	72.0	1.3	10.8	137.6	17.2	53.3	249.3	31.2	64.1	79.8	5.0
Total	5426	100	5426	100	5426	100									

*WL* woodland, *BL* bushland, *GL* grassland, *SGR* shrubby grassland, *BAL* bareland, *CUL* cultivation, *SET* settlement, *CA* changed area, *CE* changed extent, *CR* annual rate of change

LULC change detection and the transitional matrix for each LULC are presented in Table 3. Our results showed that between 1987 and 2003, woodland (159.97 km^2^), bushland (246.9 km^2^), cultivated (42.71 km^2^), and settlement land (64.10 km^2^) covers were the net LULC change gainers, whereas grassland (−177 km^2^), shrubby grassland (−334.03 km^2^), and bareland (−1.95 km^2^) were the net change losers in the transition matrix (Table 3). In the study area, bushland cover (246 km^2^) was the highest net change gainer among the various land cover types followed by woodland (159.97 km^2^). The results of this study further showed that bushland cover was expanding at the expanse of grassland, shrubby grassland, and woodland covers. On the other hand, bushland areas were converted to farmland and settlement areas. Our results displayed that shrubby grasslands were converted to a grassland cover within a landscape, while shifts were observed in all land cover classes. The shrubby grassland was entirely increased due to shifts in grassland, while mainly converted to woodland and bushland covers. Grasslands were converted to all types of LULC classes, except the woodland cover. On the other hand, our results showed that grassland was increased at the expense of woodland. Our result also showed that the bareland cover was converted to cultivation, settlement, and bushland. On the other hand, comparable size of bareland area was gained due to shifts in grassland and woodland covers. We further found that the size of cultivated land was increased due to changes in all land cover types. Likewise, settlement areas expanded at the expanse of woodland, bushland, grassland, and shrubby grassland.

**Table 3:** LULC change transition matrix showing major change in landscape (km^2^) in Borana, southern Ethiopia between 1987 and 2003

From initial state (1987)	To final state (2003)							
	WL	BL	GR	SGL	BAL	CU	SET	Total 2003
WL	**389.23**	311.72	342.15	304.79	19.93	1.32	0.29	1369.46
BL	354.26	**366.81**	524.89	167.09	28.42	1.88	0.61	1443.98
GR	388.61	361.29	**1166.89**	39.63	22.59	9.69	3.69	1994.39
SGR	35.71	94.71	55.41	**178.35**	13.35	1.30	0.19	379.03
BAL	21.13	27.42	30.19	13.36	**14.58**	1.00	0.49	108.18
CU	4.59	13.75	27.15	7.26	5.75	**0.46**	0.32	59.29
SET	15.93	21.43	25.37	2.56	5.49	0.91	**2.27**	71.96
Total 1987	1209.49	1197.12	2172.07	713.06	110.14	16.58	7.86	**2118.6 (39 %)**
Gain	980.21	1077.17	827.51	200.68	93.59	58.82	71.7	
Loss	820.24	830.31	1005.18	534.71	95.55	16.11	7.59	
Net change	159.97	246.86	−177.67	−334.03	−1.95	42.71	64.1	
Np	0.40	0.67	−0.15	−1.87	−0.13	92.85	28.24	

*WL* woodland, *BL* bushland, *GR* grassland, *SGR* shrubby grassland, *BAL* bareland, *CU* cultivation, *SET* settlement, *Net change* gain-loss, *Np* net change-to-persistence ratio (i.e., net change/diagonals of each class, in ratio)

Table 3 presents persistence as the bolded diagonal elements representing the proportions of each land use type and cover class that were static (persisted) between 1987 and 2003. As persistence is closer to the value of ‘zero,” it is an indication of a higher tendency to persist rather than decline or increase. Our results showed that the grassland cover type was the most persistent vegetation among all vegetation components followed by the woodland and bushland covers. The shrubby grassland cover was a type of vegetation component with the lowest persistence (Table 3). Overall, the present results showed that 39 % (i.e., sum of the diagonal elements) of the total landscape remains unchanged, while the remaining 61 % of the landscape were changed. The net change-to-persistence ratio (i.e., the net change/diagonals of each class) is important to indicate the dominant trends in a changing landscape. As a result, the shrubby grassland, grassland, and bareland showed a negative trend, whereas the woodland, bushland, cultivated, and settlements showed a positive trend (Table 3). The net change-to-persistence ratio of the cultivated and settlement areas was the highest, while that of bareland was the lowest (Table 3).

### Change in landscape and class level

The results of the spatial matrices at the landscape and class levels are presented in Tables 4 and 5. Our results showed that the numbers of patches were substantially increased by 19.5 % and changed to the different patches between 1987 and 2003 (Table 4). Generally, the numbers of patches were increased in the bushland (28.0 %), shrubby grassland (63.6 %), cultivated (488.8 %), and settlement (343.2 %) land cover types, while the patch numbers were reduced in other land cover types (Table 5). More patches were observed in the bushland cover type than other forms of land cover types, while the lowest patches were recorded in the cultivated land cover class (Table 5). Patch density (PD), which refers to the number of patches per 100 ha, was increased by 19.45 % between 1987 and 2003 (Table 4). The highest PD was recorded with the bushland cover, which ranged from 7.87 number of patches/100 ha in 1987 to 10 patches/100 ha in 2003 (Table 5). The largest patch index (LPI) over the last 16 years was reduced by half from 40 % in 1987 to 21 % in 2003 (Table 4). The LPI was reduced in the case of woodland (59.2 %), bushland (49.0 %), grassland (45.9 %), and shrubby grassland (53.3 %) cover classes, while the LPI was generally increased in bareland (1080 %), cultivated land (4466.7 %), and settlement landscapes (200 %) (Table 5). The grassland cover type had the highest LPI but reduced from 18.71 % in 1987 to 10.13 % in 2003 over the last 16 years (Table 5). The edge density (ED) was increased rapidly in the cultivated, settlement, and bushland cover classes, but decreased in the woodland, grassland, and shrubby grassland types (Table 5).

**Table 4:** The calculated landscape matrices at the landscape level for the Borana rangelands, Ethiopia

Parameters	1987	1995	2003	Change between 1987 and 2003 (%)
Number of patch (NP)	265,102	307,226	316,725	19.5
Patch density (PD) (no./100 ha)	28.94	33.54	34.57	19.5
Largest patch index LPI (%)	40.02	35.52	21.77	45.6
Patch richness (PR)	7	7	7	0
Shannon’s diversity index (SHDI)	1.51	1.53	1.53	1.3
Shannon’s evenness index (SHEI)	0.73	0.74	0.74	1.4
Landscape shape index LSI (m)	196.14	238.41	246.11	25.5
Mean patch fractal dimension (FRAC-MN)	1.03	1.04	1.03	0
Mean shape index (SHAPE_MN)	1.17	1.18	1.20	2.6
Mean nearest-neighbor distance (ENN_MN) (m)	93.48	87.48	91.78	1.8
Class area (CA) (ha)	542,630.1	542,630.07	542,630.07	0

**Table 5:** The calculated landscape matrices at the class level in the Borana rangelands of southern Ethiopia

Parameters	Year	WL	BL	GR	SGR	BAL	CU	SET
NP (No)	1987	70,459.00	72,071.00	53,832.00	35,656.00	24,309.00	2127.00	6648.00
	1995	53,313.00	98,990.00	52,766.00	64,240.00	14,722.00	12,963.00	10,232.00
	2003	64,864.00	92,256.00	37,654.00	58,335.00	21,626.00	12,523.00	29,467.00
Change between 1987 and 2003 (%)		−7.9	28.0	−30.1	63.6	−11.0	488.8	343.2
PD (no./100 ha)	1987	7.69	7.87	5.88	3.89	2.65	0.23	0.73
	1995	5.82	10.81	5.76	7.01	1.61	1.42	1.12
	2003	7.08	10.07	4.11	6.36	2.36	1.36	3.20
Change between 1987 and 2003 (%)		−7.9	28.0	−30.1	63.5	−10.9	491.3	338.4
LPI (%)	1987	5.25	8.03	18.71	4.05	0.20	0.03	0.01
	1995	7.46	3.44	7.80	1.02	0.06	0.02	0.01
	2003	2.14	4.10	10.13	1.89	2.36	1.37	0.03
Change between 1987 and 2003 (%)		−59.2	−49.0	−45.9	−53.3	1080.0	4466.7	200
ED (m/ha)	1987	52.43	42.96	51.09	20.92	7.95	0.62	1.60
	1995	50.40	57.70	54.84	34.88	6.93	3.01	2.11
	2003	49.38	71.52	44.76	7.56	7.76	4.52	7.56
Change between 1987 and 2003 (%)		−5.8	66.5	−12.4	−63.9	−2.4	629.0	372.5
LSI (No)	1987	345.21	284.36	250.99	179.37	173.30	50.23	89.55
	1995	300.10	388.77	286.15	300.74	151.72	130.05	111.37
	2003	305.49	430.97	229.5	222.20	170.67	134.49	203.81
Change between 1987 and 2003 (%)		−11.6	51.6	−8.6	23.9	−1.5	167.7	127.6
FRAC_MN	1987	1.04	1.04	1.04	1.03	1.03	1.03	1.02
	1995	1.03	1.03	1.03	1.02	1.01	1.4	1.21
	2003	1.04	1.04	1.04	1.03	1.03	1.03	1.03
Change between 1987 and 2003 (%)		0	0	0	0	0	0	0.98
ENN_MN (m)	1987	81.5	85.16	81.20	106.73	124.90	183.16	222.53
	1995	82.25	78.63	78.88	87.03	113.84	128.67	149.69
	2003	83.49	67.01	83.49	100.19	116.89	136.73	114.99
Change between 1987 and 2003 (%)		2.4	-21.3	2.8	−6.2	−6.4	−25.3	−48.3
SHAPE_MN	1987	1.21	1.19	1.20	1.16	1.15	1.12	1.10
	1995	1.20	1.22	1.24	1.10	1.08	1.20	1.22
	2003	1.22	1.25	1.22	1.14	1.17	1.19	1.13
Change between 1987 and 2003 (%)		0.8	5.0	1.7	−1.8	1.7	6.3	2.7
CA (ha)	1987	120,948.48	119,711.88	217,206.45	71,305.83	11,013.66	1632.12	1135.71
	1995	147,892.95	115,518.60	192,523.95	70,531.11	10,907.73	2801.43	1867.86
	2003	136,945.71	144,398.07	199,439.91	37,903.14	10,818.27	5928.66	7196.31
Change between 1987 and 2003 (%)		13.2	20.6	−8.2	−46.8	−1.8	263.3	533.4
PLAND (%)	1987	22.29	22.06	40.03	13.14	2.03	0.3	0.21
	1995	27.28	21.31	35.52	13.01	2.01	0.52	0.4
	2003	25.24	26.61	36.75	6.99	1.99	1.09	1.33
Change between 1987 and 2003 (%)		13.2	20.6	−8.2	−46.8	−2.0	263.3	533.3

*NP* number of patches, *PD* patch density, *ED* edge density, *LPI* largest patch index, *LSI* landscape shape index, *FRAC_AM* mean patch fractal dimension, *SHAPE_MN* mean shape index, *ENN_MN* mean nearest neighbor distance, *CA* class area, *PLAND* percentage of landscape, *WL* woodland, *BL* bushland, *GR* grassland, *SGR* shrubby grassland, *BAL* bareland, *CU* cultivation, *SET* settlement

Over the last 16 years, the Shannon’s diversity index (H´) for the landscape diversity was 1.52. Patch richness and Shannon evenness index (SHEI) showed negligible differences throughout the study period (Table 4). The results of our study showed that the landscape shape index (LSI) was increased by 25.5 % over the last 16 years from 196.14 m in 1987 to 264.11 m in 2003 (Table 4). We confirmed that LSI was increased in some land use cover classes with the exception of woodland (11.6 %), grassland (8.6 %), and bareland (1.5 %) (Table 5). Another shape index parameter such as mean patch fractal dimension (FRAC-MN) values of the three images showed a negligible difference and a value greater than one in all land use classes (Tables 4 and 5). The mean nearest-neighbor distance (ENN_MN) measures the average edge-to-edge distance from a patch to the nearest neighboring patch of the same type. Our results showed that the ENN_MN value was decreased from 93.48 m in 1987 to 87.48 m in 1995 and slightly increased after 1995 by 91.78 m (Table 4). We also found that the value of ENN_MN was decreased in most land cover classes with the exception of woodland (2.4 %) and grassland (2.8 %) (Table 5). Furthermore, the mean shape index (SHAPE_MN) showed that all the different classes had a value of greater than one (Tables 4 and 5). The results showed that the class area (CA) is a measure of the landscape composition, where grassland was the dominate composition in the landscape, but decreased gradually over the last 16 years (Table 5). On the other hand, woodland, bushland, cultivated and settlement land covers were increased between 1987 and 2003. These results were further illuminated by the percentage of landscape (PLAND) (Table 5).

### Underlying cause for LULC changes

**Fig. 5:**
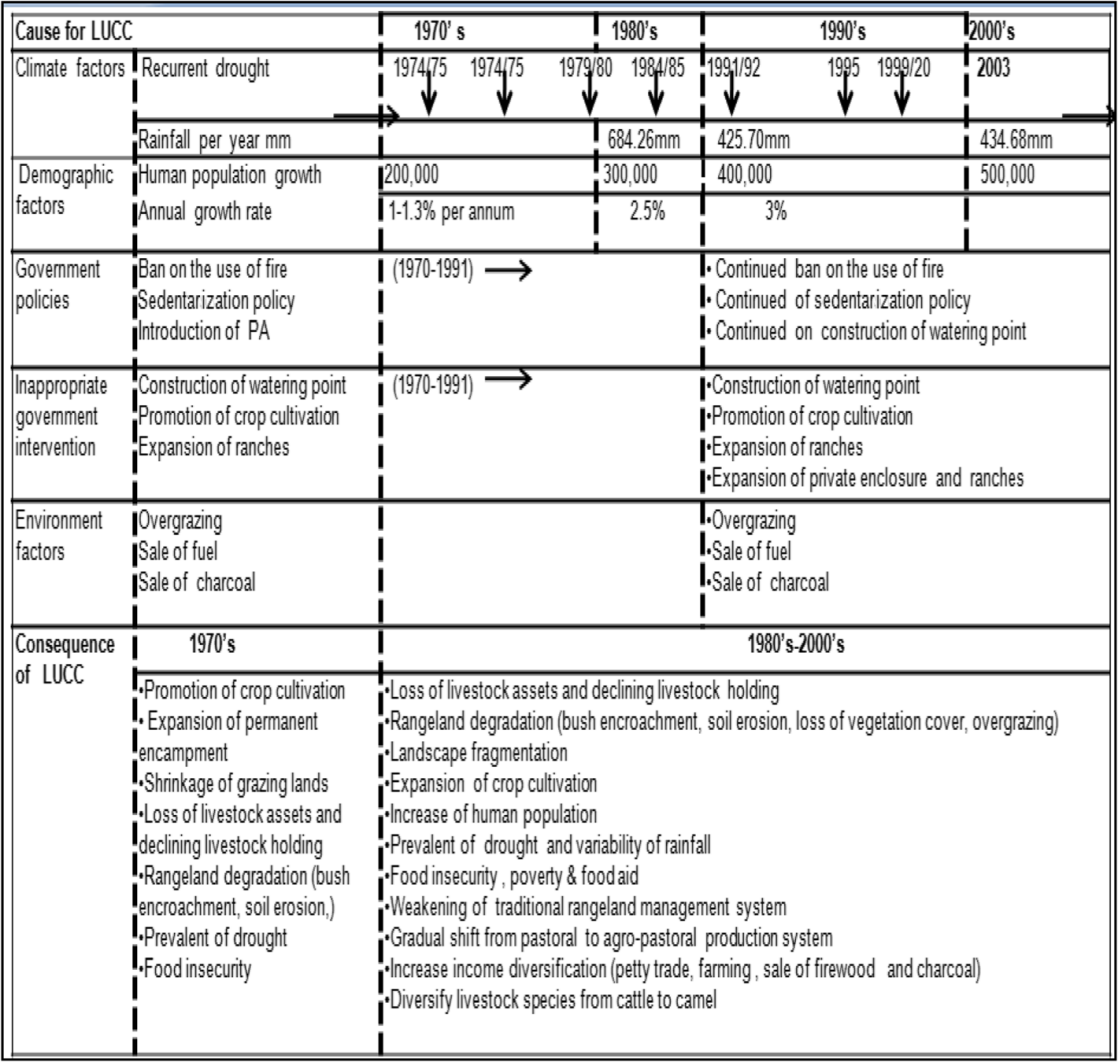
The underlying causes of LULC changes and consequences in the Borana rangelands of southern Ethiopia

Based on the opinion of the Borana pastoralists during group discussion, the main events responsible for LULC changes and associated consequences are presented in Fig. 5. Respondents mentioned the following elements as the main drivers of LULC changes, which included (i) climate-related factors such as increased temperature, recurrent drought, and rainfall variability (Fig. 5). A long-term rainfall data over the last 25 years indicate that the intra- and inter-annual rainfall distribution was highly variable. The total amount of rainfall received in 1995 (323.6 mm) and 2003 (377.1 mm) was lower than that of 1987 (649 mm). Similarly, the total amount of annual rainfall received in the 1980s (684.26 mm) was declined to 425.26 mm in the 1990s and 434.68 mm in 2000s; (ii) demographic factors (i.e., increased growth of human population from 200,000 in the 1970s, 300,000 in the 1980s to over 500,000 in the early 2000s (Fig. 5); (iii) implementation of inappropriate government policies (i.e., ban of fire, promotion of crop cultivation, settlement policies, and introduction of peasant association); (iv) inappropriate development interventions such as development of water points, allocation of land to ranches, private investors, and appropriation of private pasture lands; and (v) anthropogenic factors such as increased grazing pressure, sale of firewood and charcoal extraction. Pastoralists mentioned that the impact of LULC changes resulted in rangeland degradation, which was explained in terms of bush encroachment, increased grazing pressure, loss of herbaceous vegetation cover, and soil erosion. Another consequence of LULC changes included the fragmentation of individual landscapes (Teshome 2011), reduced grazing capacity, decline in livestock holding per pastoral household, and loss of livestock asset. Consequently, poverty and food insecurity, weakening of the traditional institution in natural resource management practices, and looking for alternative livelihood income and diversification (i.e., promotion of cultivation, petty trade, and rearing of the different livestock species for instance camel) were some of the associated impacts of LULC changes in the study area.

## Discussion

### Magnitude of LULC changes

The Borana rangelands in southern Ethiopia have experienced extensive and escalating rates of LULC changes between 1987 and 2003. The present findings provide a wealth of information on LULC patterns in the study area. Over the last 16 years, extensive land cover changes have taken place, both spatially and temporally. The observed increase in bushland cover is in agreement with previous reports (Coppock 1994; Solomon et al. 2006b; Dalle et al. 2006; Angassa and Oba 2008a; Haile et al*.*^2010^). According to Assefa et al*.* (1986), about 40 % of the Borana rangelands were affected by bush encroachment in the 1980s, while Dalle et al. (2006) have estimated the progression of bush cover to 52 %, which is in agreement with the present result (i.e., the sum of bushland and woodland covers together count for 52.85 %). A study by Mesele et al. (2006) reported a 15 % increase in bushland cover between 1986 and 2002, which is comparable with the results of the present finding (i.e., 17 % increase in the bushland cover). The possible explanation for the increase in bushland cover due to the ban of fire as a tool to control bush encroachment and continued human disturbances linked to over grazing practices. On the other hand, excessive human exploitation of the woody plants for different purposes probably facilitated the decline in woodland cover between 1995 and 2003.

As observed in the study area, grassland cover was the dominant LULC type between 1987 and 2003. However, a progressive decline in grassland was observed due to the various driving forces (i.e., bush encroachment, drought, expansion of cropland, settlement, construction of water points). Our results are generally in agreement with the report by Haile et al. (2010) which reported that use of aerial photograph of 1987 and Landsat 2002 grassland cover declined by 8 %, which is comparable with the present result (7.73 %). Other sources (e.g., Coppock 1994; Dalle et al. 2006; Solomon et al. 2006a; Angassa and Oba 2008b) have also indicated that the extent of grassland cover was greatly reduced.

The observed low proportion of settlement areas as compared to other land use types in the present study could be settlement patterns that were scattered, heterogeneous, and too small in size to be detected by the Landsat TM. This might also underestimate the status of settlements in the Borana pastoral areas and considered as a limitation in terms of the application of Landsat image in such environment. Conversely, the observed increase in the size of settlement areas during the last 16 years could be linked to the increase in human population growth following the development of semi‐permanent water points in the 1970s (Angassa and Oba 2008b) and settlement policy of the Ethiopian government between 1970 and 1991 (Oba 1998).

The low proportion of cultivated lands in the study area is most likely associated with the past robustness of the Borana social institution that prohibits cultivation in the rangelands (Angassa and Oba 2008b). Additionally, the expansion of cultivation is a recent phenomenon in the Borana rangelands due to inappropriate development interventions and weakening of the local institution. Furthermore, the size of individual farm lands is small and less than a hectare. Such small fields are scattered in pattern and difficult to be detected by Landsat TM. Thus, such condition might also underestimate the total cover of cultivated areas in the present study. According to Coppock (1994), in the 1980s, about 45 % of the pastoral households were involved in crop cultivation, while the average cultivated plot size per household was 0.29 ha. Angassa and Oba (2008b) have indicated a more recent expansion of crop cultivation in the Borana rangelands with 80 % of the crop fields that were less than 20 years old. Although the total size of cultivated land area was relatively small, increased participation in crop cultivation has been observed since the last 16 years. This suggests that the Borana pastoral way of life is gradually shifting from more dependence on livestock keeping to crop cultivation in some locations. Pastoral communities are forced to involve in crop farming to meet their families’ food demand subsequent to repeated drought and massive loss of livestock, lack of land use policy, and government’s crop-focused agricultural priority via increased access to improved seed, fertilizer, and farm implements (Angassa and Oba 2008b). The increase in cultivated land cover at the expense of grassland in the study area reflect that crop farming has undertaken mainly in bottomland areas where seasonally flooded rangelands and moisture conditions are favorable and dominated by vertisols. Bottomlands are key areas for calf grazing reserves for the dry season use (Coppock 1994). Generally, the loss of bottomland areas to crop farming may exacerbate the vulnerability of livestock especially calves to drought (Angassa and Oba 2007; Angassa 2011).

### Change in landscape structure

The Borana rangelands have undergone considerable alterations between 1987 and 2003. The observed increase in the numbers of patches, which refers to a simple measure of the extent of fragmentation of the patch type over years (McGarigal et al. 2002), demonstrating the breaking up of individual landscapes into smaller parcels and the number of patch often leads to reduction of the overall size of rangeland habitat, shrinking of the core area, reduction in the size of the largest patch, increase in patch density, and isolation of the habitat. Patch density is simply a measure of landscape fragmentation (Thapa and Murayama 2009; McGarigal et al. 2002) and has increased in the study area overtime. The increase in patch density also leads to an increase in the edge density due to the creation of new edge segments in the patch (Thapa and Murayama 2009; Mhangara and Kakembo 2012), and the increased edge reduced the interior of core area of land cover. The increased edge has ecological implication on native plant and animal. These landscape matrices clearly illustrate an increase in grassland fragmentation over the study period, and similar trend was observed in other parts of the world (Herold et al. 2003; Turan et al. 2010; Sidiropoulou et al. 2015; Mhangara and Kakembo 2012). This indicates that there is an extensive land use changes in Borana rangelands between 1987 and 2003, which have probably occurred as a result of various factors. This is further supported by the largest patch index, which measures fragmentation and dominance in the landscape (McGarigal et al. 2002) that could be reduced overtime. The size of the LPI for vegetation component within a landscape has decreased as a result of fragmentation. The fragmentation of a landscape is more visible in the grassland cover type. Increased in grassland fragmentation negatively affect grazing animals and leads loss of grassland diversity and ecological stability. The observed dominance of the grassland cover in individual landscapes is further illuminated by the PLAND, which quantifies the proportional abundance of each patch class within individual landscapes. The present finding indicates that between 1987 and 2003, the H´ value for the landscape diversity was 1.52, which implies that the individual landscapes had maintained heterogeneity. Furthermore, the observed value for SHEI in the current study illustrates irregular distribution of patches within a landscape. The FRAC-MN usually describes the complexity and fragmentation of patch dynamics; and it approaches the value of one for shapes with very simple perimeters such as circles or squares; it approaches two for shapes with highly convoluted and plane-filling perimeters (McGarigal et al. 2002). The observed value in our study is greater than the value of one. This implies that the average patch shape in all landscapes is nonsquare and irregular in shape, suggesting that an increase in shape complexity is an indication of the presence of anthropogenic disturbance (Krummel et al. 1987). The shape of a patch can influence important ecological processes in the landscape such as changes in microclimate and disturbance factors (Leitao and Ahern 2002). The observed ENN_MN values simply indicate the inter-patch proximity and isolation due to landscape fragmentation (McGarigal et al. 2002). This implies that reduction of the distance between neighboring patches of similar land use was as a result of formation of numerous new patches within a close proximity thereby contributing to the processes of fragmentation that include: bush invasion, construction of water points, expansion of cropland and settlement in grasslands. The declining mean nearest-neighbor distance for bush lands as compared to other vegetation land cover of the rangeland could be expansion of bush plant in close proximity to existing habitat patches of bushland. Therefore, patch separation in the Borena grassland is the result of anthropogenic effects. In general, the change in LULC has resulted in landscape fragmentation, which is indicated by the proliferation of large numbers of patches by increasing patch density and decreasing the largest patch index and irregular shape of patch within individual landscapes and at each class level. With the concept of landscape fragmentation (i.e., the breaking up of habitat or land type into smaller parcels), there are other spatial processes in land transformation that need to be mapped: perforation, dissection, shrinkage, and attrition (Forman and Godron 1986). There are many open savanna grassland vegetation patches that decreased in size, disappeared, or become perforated. For instance, the construction of water points in the former wet season grazing areas, establishment of ranches, promotion of cultivation, sedentarization, expansion of bushland, and recurrent drought in the region since the 1980s, all these, contributed to the subdivision of certain vegetation patches into sections (Reid et al. 2004; Dalle et al. 2006; Angassa and Oba 2008b). Therefore, this kind of landscape information may highlight and support the claim that the Borana rangelands have been degraded. Considering LULC dynamics in the Borana rangelands, it is likely that the landscape change tendency in the future might continue.

### Underlying cause for LULC changes

The underlying causes and associated consequence are explained in terms of the major events (socio-ecological, environmental, policy and development intervention) that took place in the study area since the 1970s. We considered climate change-related events (rainfall variability, recurrent drought, and temperature), development intervention-related events (i.e., promotion of ranch, crop cultivation, private enclosure, construction of water point), policy-related events (i.e., ban of fire, promotion of crop cultivation, settlement, introduction of peasant association), and the observed changes in LULC and vegetation dynamics within individual landscapes influenced by the direct and indirect effect of drought. Opdam et al. (2009) suggested that the impact of climate change will affect all types of land use and ecosystem services, as well as the behavior of humans. The impact of drought in Borana has been widely documented (Oba 1998; Desta and Coppock 2002; Homann et al. 2005; Angassa and Oba 2008b; Tache and Oba 2010). Multiple droughts most likely affect vegetation cover mainly by suppressing the grass cover, while stimulating bushland/woodland cover. These phenomena can contribute to a decline in grassland and shrubby grassland cover and alter the original landscape and habitat for grazers. Furthermore, periodic drought in the Borana rangelands of southern Ethiopia could induce chronic poverty and local food insecurity (Tache and Oba 2010) that associated with rangeland degradation (Dalle et al. 2006; Solomon et al. 2006b; Angassa and Oba 2008b) and massive loss of livestock (Cossins and Upton 1988; Desta and Coppock 2002; Angassa and Oba 2007). In the study area, drought brought promotion of crop farming at the expanse of the communal rangelands, expansion of bush encroachment that reduces mobility, utilization of woodland vegetation, weakening of the traditional rangeland management practices, and decline in livestock mobility during drought periods. To explicitly understand the impacts of drought in the region, climate change might have a cofounding effect with overgrazing, population pressure, ethnic conflict, and land degradation together with other factors that would lead to changes associated with LULC and landscape structure. The conversion of grassland to other land use such as cropland, bushland, and bareland can break the vast grassland vegetation patch into smaller pieces of different sizes and shapes. This clearly illustrates an increase in grassland and shrubby grassland vegetation fragmentation over the study period, as demonstrated by increase in the number of patches, patch density, and edge density that enhances isolation of patches and irregular shape of patch, while decreases the largest patch index and total core area. For example, Li et al. (2001) affirmed similar trends in their study in quantifying landscape structure in the Heihe river basin, north-west China using FRAGSTATS. Others (e.g., Reid et al. 2004; Flintan 2011; Flintan et al 2011) have reported that climate variability such as recurrent drought and rainfall variability are among the major driving forces for rangeland fragmentation and loss of habitats in East Africa rangelands. Generally, the trend suggests that the effect of recurrent drought could be more detrimental in driving LULC changes in the study area. The observed long-term rainfall data indicate that the intra- and inter-annual rainfall distribution was highly variable. The variation in the amount of inter-annual rainfall to a great extent causes differences in vegetation cover mainly by altering the grassland state to the woodland and bushland states. In arid environments, plant productivity is strongly dependent on rainfall variability (Ellis and Swift 1988). In the study area, inter-annual rainfall variability promotes the regeneration of invasive woody seedlings (Angassa and Oba 2008a), suggesting that the original grassland state was highly fragmented creating a barrier for the free movement of grazers within encroached areas. Overall, the present analyses suggest that climate variability in terms of episodic drought or variable rainfall is probably a potential driver of LULC changes and rangeland fragmentation with associated impacts on feed supply for grazing animals and food insecurity for pastoral households.

Demographic factors related to population growth are usually among the underlying causes for LULC changes in the study area as in other parts of East African savannas (Reid et al. 2004; Tsegaye et al. 2010). In the 1980s, the human population of Borana was estimated to 300,000, whereas the figure has increased to over 500,000 in the early 2000s (Oba 1998; Homann et al. 2005; Mesele et al. 2006). Furthermore, the annual human population growth rate in Borana was about 1–1.3 % in the early 1970s (Homann et al. 2005), but increased to 2.5 % in the 1980s (Coppock 1994). In the late 1990s, the net population growth was estimated at 3 % per year (Helland 1997). The continuous increase in human population brought substantial changes in terms of the expansion of cultivation and settlements and intensifies severe pressure on the existing rangeland resources (i.e., use of woody plants for construction purposes, fuel wood, and charcoal). This contributes to the changes in LULC and landscape fragmentation. Earlier studies (Dalle et al. 2006; Solomon et al. 2006b) have reported that the increase in population pressure might be one of the causes of rangeland degradation. Demographic-related variable as evidenced in other parts of East African countries (Flintan et al 2011; Flintan 2011) is also among the causes for landscape fragmentation and loss of rangeland habitats. From this, one can draw that the growth of human population together with other anthropogenic factors could contribute to the shifts in LULC and landscape fragmentation.

Government policies including ban on the use of fire and expansion of cultivation and settlement programs in the rangelands were reported to be the main causes for LULC changes (Oba 1998; Coppock 1994; Homann et al. 2005; Angassa and Oba 2008b). Fire is a key ecological factor that regulates the normal functioning of savanna ecosystems (Bloesch 1999). Furthermore, fire plays an essential ecological role in shaping the structure and composition of rangeland vegetation (Laris 2002; Angassa and Oba 2008b). The Borana pastoralists traditionally used fire as a tool for rangeland management by controlling the expansion of bush encroachment (Oba 1998). However, the use of fire as a tool for rangeland management was prohibited in the early 1970s (Coppock 1994). As a result, the bushland and woodland covers have significantly increased by taking the competitive advantages of tree seedlings over grasses (Angassa and Oba 2008a). The observed increase in bushland at the expense of grassland and shrubby grassland may support the hypothesis that suppression of fire as a management tool stimulates the bushland cover (Oba 1998; Angassa and Oba 2008b; Angassa et al. 2012a). The observed shifts from grassland to bushland cover in the study area can occur if grass is overgrazed beyond its capacity to recover quickly or following a drought that might stimulate tree seedling development (Oba 1998). The ban of fire might contribute to the progressive increase of bush and woody plants cover (Oba et al. 2000; Angassa and Oba 2008a). These policy-driven activities have adversely affected the changes in LULC and facilitates the processes of bush encroachment as a driving force in the fragmentation of grassland and shrinkage in the capacity of rangelands for grazing.

Since the 1970s, many development projects have been widely implemented in the Borana rangelands of southern Ethiopia (Oba 1998; Coppock 1994; Homann et al. 2005). Inappropriate development interventions such as allocation of grazing lands to government and private ranches greatly reduced livestock mobility and the status of grassland cover (Oba and Kotile 2001). Furthermore, following the development of water points in the 1970s, human settlements were attracted in the vicinity of permanent water points. As a result, between the 1970s and 1990s, the number of settlements was increased by more than 200 % (Oba 1998). In some areas, the pressure on pastoral land use as a result of the creation of unplanned water points led to heavy grazing pressure forming bareland areas that contributed to the wide spread degradation and fragmentation of rangeland resources (Coppock 1994; Oba and Kotile 2001; Solomon et al. 2006b), which may also be contributed to the increase in bareland cover. In the Borana region, mobility and opportunistic resource utilization are key management strategies attributed to the uncertainty of climate. However, inappropriate development interventions contributed to the restriction of the free movement of livestock in the study area, thereby accelerating land degradation. In the study area, over 10,000 ha of grazing lands were allocated to ranches (Oba and Kotile 2001). Most importantly, the areas allocated to ranches were the best parts of the rangelands. Loss of key grazing areas and inappropriate water sources have exacerbated rangeland degradation, and this has resulted in the conversion, modification, and fragmentation of the communal rangelands that were purely used for grazing by the pastoral communities. Previous studies in East African rangelands (Flintan et al. 2011; Flintan et al. 2011) have indicated that the establishment of ranches and private enclosures are the main causes for the fragmentation of rangeland habitats. Oba and Kotile (2001) further argued that ethnic conflicts over disputed resources and displacement of pastoralists from their traditional grazing lands might intensify the process of rangeland deterioration due to increased grazing pressure on the remaining portion of rangeland resources.

Inappropriate policies and misconceptions of traditional resource management have led to the expansion of bush encroachment and reduced grazing capacity of the communal rangelands in the study area. Furthermore, the imposition of Peasant Association (PA) since the 1970s for political administration at the local level has resulted in the weakening of the traditional institution (i.e., *Gada* system) and communal rangeland management practices. The promoted Peasant Association has taken over the decisional roles of pastoral elders and further disturbed the networks by which the Borana pastoralists used to govern access to pastures and water points. Such policy approach restricted access to the communal grazing lands and mobility between the dry and wet season grazing areas by weakening the traditional rangeland and water management practices. The new approach further facilitated sedentarization and adoption of private enclosure. Overall, policy-driven changes such as the official ban of range burning for pasture improvement, expansion of bush encroachment, establishment of ranches, promotion of crop cultivation, and the emergence of semi-private enclosures significantly contributed to landscape fragmentation and severe rangeland degradation.

The observed changes in LULC in the Borana rangelands have resulted in a substantial change in livestock holding at the household level, while communities in general lost their livestock asset and become destitute. This might further be linked to the combined effects of shifts in grassland cover and recurrent drought (Angassa and Oba 2007). Another contributing factor for LULC changes are the process of bush encroachment linked to rangeland degradation and fragmentation (Dalle et al. 2006; Angassa and Oba 2008b). Bush encroachment is widespread since the last four decades with its negative effects on cattle production and pastoral communities’ livelihood (Angassa and Oba 2008b). In the Borana rangeland, grazing resources have been owned communally and administered by the traditional institution where community leaders used to formulate bylaws about their use with respect to the spatial and temporal patterns of grazing and types of animals to be allowed to graze in a specific landscape (Oba 1998). Changes in LULC have weakened the traditional institution in resource management. As a result, the present condition of resource administration and management in terms of rangeland resource utilization is not as strong as some four decades ago. Overall, the trends in LULC changes can adversely affect rangeland resources and livelihood of the local people.

## Conclusions

The results of our findings are relevant for the development of appropriate land use policy in the arid and semi-arid regions of Ethiopia. The results showed considerable changes in LULC patterns both spatially and temporally. We found that grassland and shrubby grassland covers were in a declining trend between 1987 and 2003 (last 16 years). On the other hand, the size of woodland, bushland, settlement, and cultivated land covers were considerably increased. As a result, the conversion of grassland and livelihood diversification from a livestock dominated production system to crop cultivation is an emerging land use change in the region. The results further showed that the density of bush encroachment was increased at the expense of grassland thereby reducing the capacity of grasslands for cattle production.

Landscape fragmentation is also an important issue as a result of LULC changes in the Borana rangelands of southern Ethiopia. Our findings indicate that the original grassland state was fragmented due to the formation of large number of small patches and increased patch density. Consequently, the fragmentation of a vast area of grasslands into small patches may support the claim that the Borana rangeland is gradually degraded and reduced in size. It seems that the underlying causes of LULC changes are mostly attributed to the impact of rainfall variability and recurrent drought, population growth, and inappropriate government policies for pastoral area development and other anthropogenic factors. Changes in LULC greatly contributed to rangeland degradation and may lead to the loss of Borana cattle breed in the region. Our results suggest that continued LULC changes as a result of man-made and natural disasters might adversely affect the local environment and livelihood of pastoral communities. The increase in rangeland fragmentation include loss of grasslands through conversion to bush-dominated landscape and cultivated lands, which is threat to native plant diversity, community, and livestock production. The fragmentations led to decline in grass cover and restrict seasonal herd mobility.This trend contributes to the reduced resilience and high vulnerability of the Borana pastoralists to food insecurity. Therefore, the urgency of measures to halt rangeland fragmentation is recommended. Analyses of landscape structure through spatial matrices were found to have considerable practical value for management of arid and semi-arid rangelands of Borena.

## Footnotes

1 Presence of well clusters sunk in limestone rocks that represented the active and disused *tula*-wells. The word “tula” in the Borana Oromo language means high yielding—usually in reference to water. The *tula-*well complexes represent an ancient source of water used by the pastoral community in southern Ethiopia for several hundred years. The ancient *tula*-well complexes are special savanna cultural landscapes in southern Ethiopia. The *tula-*wells play a vital role in the Borana society in the management and preservation of the grazing landscapes, cultural identity, and a focal point for social organization and ritual practice (Tiki et al. 2011).
